# Impact of child disability on parental employment and labour income: a quasi-experimental study of parents of children with disabilities in Norway

**DOI:** 10.1186/s12889-022-14195-5

**Published:** 2022-09-24

**Authors:** Michael Yisfashewa Wondemu, Pål Joranger, Åsmund Hermansen, Idunn Brekke

**Affiliations:** 1 Norwegian Social Research, Section for Health and Welfare Research, Oslo Metropolitan University, Oslo, Norway; 2 Faculty of Health Sciences, Department of Nursing and Health Promotion, Oslo Metropolitan University, Oslo, Norway; 3 Faculty of Social Sciences, Department of Social Work, Child Welfare and Social Policy, Oslo Metropolitan University, Oslo, Norway; 4 Department of Child Health and Development, Norwegian Institute of Public Health, Oslo, Norway

**Keywords:** Children with disabilities, Indirect costs, Parental employment, Difference-in-differences design

## Abstract

**Background:**

Caring for children with disabilities has both immediate and long-term economic costs that affect the well-being of children, parents, and society. The purpose of this study was to investigate the impact of child disability on parental employment and labour income by examining differences by parental gender, disability severity, and child age.

**Methods:**

The study included children with disabilities born between 2004 to 2011 and their mothers (*n* = 139,189) and fathers (*n* = 134,457). Longitudinal data on employment, working hours and labour income was obtained from Statistics Norway, specifically the National Education Database, the Central Population Register and the Event History Database. A quasi-experimental difference-in-differences model was used to examine differences in employment, working hours and labour income.

**Results:**

The results showed that caring for children with disabilities has a negative effect on mothers’ labour market participation, working hours and labour income. The more severe a child’s condition is, the more likely the mother was to work and earn less, or to stop working entirely. Additionally, the differences in labour market participation and income between mothers of children with and without disabilities increased as their children reached school age. Labour market participation, working hours, and labour income for fathers of children with less severe disabilities is comparable to those of fathers of children without disabilities. Caring for children with more severe disabilities reduces fathers’ labour income but has no effect on their working hours or labour market participation.

**Conclusion:**

Policymakers and child welfare stakeholders should evaluate policy options and provide the necessary welfare support particularly to mothers caring for children with a more severe disability.

**Supplementary Information:**

The online version contains supplementary material available at 10.1186/s12889-022-14195-5.

## Introduction

The estimated proportion of children with disabilities worldwide ranges between 5 and 10%, depending on the source [1]. Disabilities in children involve a variety of immediate and long-term economic costs that have important consequences for the well-being of children, parents and society. Caring for children with disabilities involves indirect economic costs, that places a financial burden on the family [2]. An important indirect cost for these families involves decisions about employment [3].

Raising a child affects parents’ participation in labour market, especially among women [3]. Since the early twenty-first century, work-family conflict has increased as women have increasingly entered the workforce, as part of a trend that has altered the role of married men and women in caring for their children [4]. Despite increased focus on gender equality in today’s world, women remain the primary care givers of children. As a result, they face significant challenges in balancing their occupational obligations and care-related responsibilities [5, 6]. The pressure from work and care responsibilities is even stronger for parents of children with disabilities, particularly the mothers [7]. These established consequences of work-to-family conflict include decrease in labour market participation, higher risk of unemployment and drop in income level, all depending upon the severity of the disability of the child, the parent’s socioeconomic status, the environment in which they live, government policy and the corresponding welfare system [8]. The present study used longitudinal data to examine whether young children’s disabilities impact labour force participation and income for mothers and fathers.

While various studies on this area are cross-sectional analysis with small sample size or non-representative groups [9, 10], longitudinal research on trends in parental employment has been limited. Therefore, the present study examined whether trends in labour market participation and income have changed among Norwegian parents because of caring for children with disabilities. It focused on variations in parental employment, working hours and labour income based on gender, disability severity and the age of the child. Norway is an interesting case due to high employment among mothers—in 2019, 81%, among the highest in Europe—and strong national support for parents seeking to combine work and care-related responsibilities. Beyond that, the gender gap in labour force participation among parents in Norway, at less than 10%, is remarkably low.

The results of this study highlighted a negative impact of caring for children with disabilities on employment probabilities and labour income among parents, particularly for mothers. The main strength of the study was its use of high-quality register data to follow up the long-term employment effects of caring for children with disabilities until the child grew older. This provides essential insights for policymakers about the extent of the problem both on the short and long term, thereby helping parents provide the necessary welfare support to enhance their work–family life balance.

Following the introduction, the article discussed prior research and theoretical approaches to present the study hypotheses. It then explained the Norwegian welfare state and family care. After describing the study’s methodology, results, discussions, and concluding remarks were presented.

### Previous research, theoretical approach and hypotheses

International and Norwegian studies have investigated the parental employment consequences associated with disabilities among children. Such studies consistently found associations between intensified care needs and reduced labour market participation [7, 11–13]. Olsson and Hwang [12] found that parents of children with disabilities are less likely to be involved in a paid employment and tend to have lower levels of well-being. This finding was evident in Burton et al. [9] that examined the relationship between children health and mothers labour market outcomes. A study conducted in Spain by Cantero-Garlito et al. [14] demonstrated that caring for children with disabilities requires greater investments of time and resources than caring for children without disabilities. This can hamper parents’ participation in employment. We also expect that due to their children’s increased care needs, parents of children with disabilities may withdraw from or lose stable full-time employment and engage in part-time employment.

- *H1: Parents of children with disabilities are less likely to be employed.*
- *H2: Parents of children with disabilities are less likely to be in full-time employment.*
- *H3: Caring for a child with a disability will result in lower labour income.*

Studies consistently report that women tend to experience more work-family conflict than men. Hauge et al. [11] study in Norway found that many mothers face reductions in working hours or permanently withdraw from the labour market while caring for their children with disabilities. A similar pattern was found by Brekke and Nadim [7], which reported that when children need increased care, their mothers earn less, probably due to reduced participation in the workforce. Such consequences may occur because, according to specialization theory, mothers are expected to take responsibility for taking care of their children with special needs and are thus more vulnerable to the need to reduce or give up entirely on paid employment [15]. Specialization theory explains the division of labour as related to utility maximization. The main determinant for the division of paid and unpaid work among partners depend on the comparative advantage of income. Because mothers often earn less, they tend to specialize in childbearing and other domestic activities, whereas fathers engage more in the labour market [16]. This is consistent with the gender-role theory which views gender role expectations and norms socially imposed on both men and women affect work-family balance [17]. Society expects women to focus on household work and men to play the breadwinner role [18]. Therefore, according to both such theoretical approaches and the findings of previous studies, mothers’ employment and labour income should be affected more than fathers because of having children with disabilities.

- H4: *The negative employment effects are stronger for mothers than for fathers.*

Parents of children with disabilities face an increased risk of reducing their participation in or even withdrawing entirely from the labour market when the disability is severe [11, 19–21]. The severity of a child’s disability has been associated with lower levels of job satisfaction and work–family balance and higher levels of stress, which can affect parents’ participation in paid work [22]. A study by van Dyck et al. [21] in the United States found a relation between the severity of the child’s condition and parental likelihood to reduce working hours or stop working altogether. A similar pattern was found in a Norwegian study [11], which showed that children with chronic disability increase mothers’ long-term absence from work due to sickness and reduce their income. However, there were no significant differences in the likelihood of employment participation between mothers of children with less severe disability and those caring for children without disabilities; rather, mothers caring for children with less severe disabilities were more likely to reduce working hours and have part-time employment than other mothers. The present study also postulates that adverse employment effects should be stronger for parents of children with more severe disabilities.

- *H5: The negative employment effects are stronger for parents of children with more severe disability.*

Although results remain inconclusive [15], household factors such as child age and family composition may influence parental employment [22–24]. A study in Australia by Bourke-Taylor et al. [25] demonstrated that the younger a child with disability is, the more negative the effect on parental employment. Parents of school-aged children with disabilities were less likely to be affected than parents of pre-school children. Similarly, Loprest and Davidoff [24] revealed that the likelihood of parental employment reduces as the age of the child with disabilities decreases. This could be due to a scarcity of childcare facilities for children with special needs. Childcare is a major concern for parents caring of children with disabilities because they may not have all the financial resources they need, even when a care facility is provided for their children [22]. Accordingly, we proposed the following hypothesis:

- *H6*: *The negative employment effects are stronger among parents of pre-school children.*

### The Norwegian welfare state and family care

Norway is a social democratic welfare regime characterized by generous social insurance and universalism [26, 27]. It provides financial and care assistance for parents of children with disabilities. The national insurance scheme provides financial support given on a monthly basis, including basic, standard attendance and higher rate attendance benefits, to compensate for the additional costs related to intensified care needs [28, 29]. However, the support is not enough to fully compensate for the job loss. Higher rate of attendance benefits is provided for parents whose needs for care and supervision significantly exceeds than the standard attendance benefits. The provision of such support depends on the degree of impairment, the parents’ workload in providing care, and the type of care needed [30]. Municipalities and local welfare agencies provide care assistance, such as respite and institutional care. Municipalities may also pay additional support (*a care wage*) for families of children with disabilities. The care wage varies considerably across municipalities and is often paid to mothers [28]. In addition, children with disabilities receive access to day care until they reach the seventh grade, which is greater than what normally developed children receive (i.e., until fourth grade) [30]. The day care facility may help to promote employment among parents of children with disabilities.

The Norwegian welfare state has long focused on people’s participation in paid work, including mothers [27]. Since de-institutionalization of long term care for people with disabilities in late nineteenth century and the increased participation of women in the labour market, the question of how much work compensated due to care responsibilities has arisen [28]. Norway provides a generous parental leave and childcare services to ensure the participation of parents in the labour market, which in turn fosters an inclusive labour market. Parents are provided with approximately a year of paid parental leave depending on their employment status before giving birth, (i.e., 46 weeks with full wage or 56 weeks with 80% compensation). Mothers often take the largest share of the sate-sponsored leave, with 10 weeks of absence reserved for fathers, i.e., *the Daddy quota*. Sick pay scheme in Norway administered by the Norwegian Labour and Welfare Administration (NAV), also considered to be among the most generous in the world, provides all paid workers full wage compensation for up to 50 weeks, and the first 16 days of sick leave are funded by employers if the workers have been employed for at least a month [27].

### Data

The study used register data from Statistics Norway (SSB), specifically the National Education Database, the Central Population Register and the Event History Database (FD-Trygd). The FD-Trygd consists of information on parents’ age, attendance benefits, employment and labour income. Because children in Norway with long-term medical conditions often receive attendance benefits from the Norwegian Labour and Welfare Administration, children with disabilities were identified according to information about attendance benefits using FD-Trygd. Such benefits are paid at a fixed rate and granted based on the care needs of the children, independently of any other income. The study was limited to children with disabilities born between 2004 and 2011, along with their mothers (*n* = 139,189) and fathers (*n* = 134,457). The control group consisted of parents who did not have children with disabilities during the observation period. We restricted the analyses to primipara mothers. To examine trends in parental employment, we used the register’s longitudinal information about parents’ employment status beginning 4 years prior to their children’s birth up to 10 years after birth. The register data includes annual information on education, employment, working hours and income along with concise information relating to when the child was born.

The three dependent variables in the present study are labour market participation, working hours and annual labour income. Labour market participation was measured as a dummy variable and is coded 1 if the parent was employed and 0 otherwise. Parents were classified as *employed* if they worked as paid employees during the reference week (^3rd^ week of November). *Working hours* was measured as full- versus part-time employment to determine the likelihood of being employed full time. *Annual labour income* was measured as all income from paid employment annually and as a continuous variable. A logarithmic transformation was used to ensure a more normal distribution of the outcome variable.

*Having children with disabilities* was measured based on information about children who received attendance benefits during the observation period. The attendance benefits pay grades (1–4) determined by authorities was used as a proxy for severity. A dummy variable was created for having a child with versus without disabilities. *Age of parents* at birth was measured as a continuous variable in number of years. *Marital status* was measured by a dummy variable indicating whether the mother and father were married. *Immigrant background* was measured as a dummy variable differentiating between parents born abroad and those born in Norway. *Educational level* was also a categorical variable classified into four levels: no education, compulsory education, upper secondary education and any college and university. Additionally, birth cohort and the number of younger siblings born during the observation period were controlled for in the analyses.

## Methods

A quasi-experimental difference-in-differences (DiD) study design was used to examine the effect of having a child with disability on employment and labour income in the period from four years prior to ten years after birth. The DiD model compares changes in the outcome variable over time for parents caring for a non-disabled child to the changes over time for parents caring for a child with a disability. The observed differences can be attributed to the effect of caring for a child with a disability. Because data on parental employment suitable for comparison were only available for 2000, we included a sample of children from 2004 to 2011.

We estimated empirical models of the following form:

$${Y}_{it}={\beta}_0+{\beta}_1 Disabilit{y}_i+{\sum}_{t=-4}^{t=10}{\beta}_{3t}{T}_{it}+{\sum}_{t=-4}^{t=10}{\beta}_{4t}{T}_{it} xDisabilit{y}_i+{\beta}_5{X_i}_t+{\varepsilon_i}_t$$

Subscript i refers to individuals and t to time. Disability grades (1–4) is a variable which represents parents caring for a child with a disability. T is a vector of t-1 time fixed effects varying from four years prior and ten years after birth. Disability T is a vector of t-1 interaction terms between disability grades (1–4) and t-1 year fixed effects. X is a vector of individual-level control variables. Our key interest is in the β values for Disability T. These coefficients tell us how the difference between having a child with a disability (grade 1 to 4) and having a non-disabled child develops over time, relative to a reference period.

**Fig. 1:**
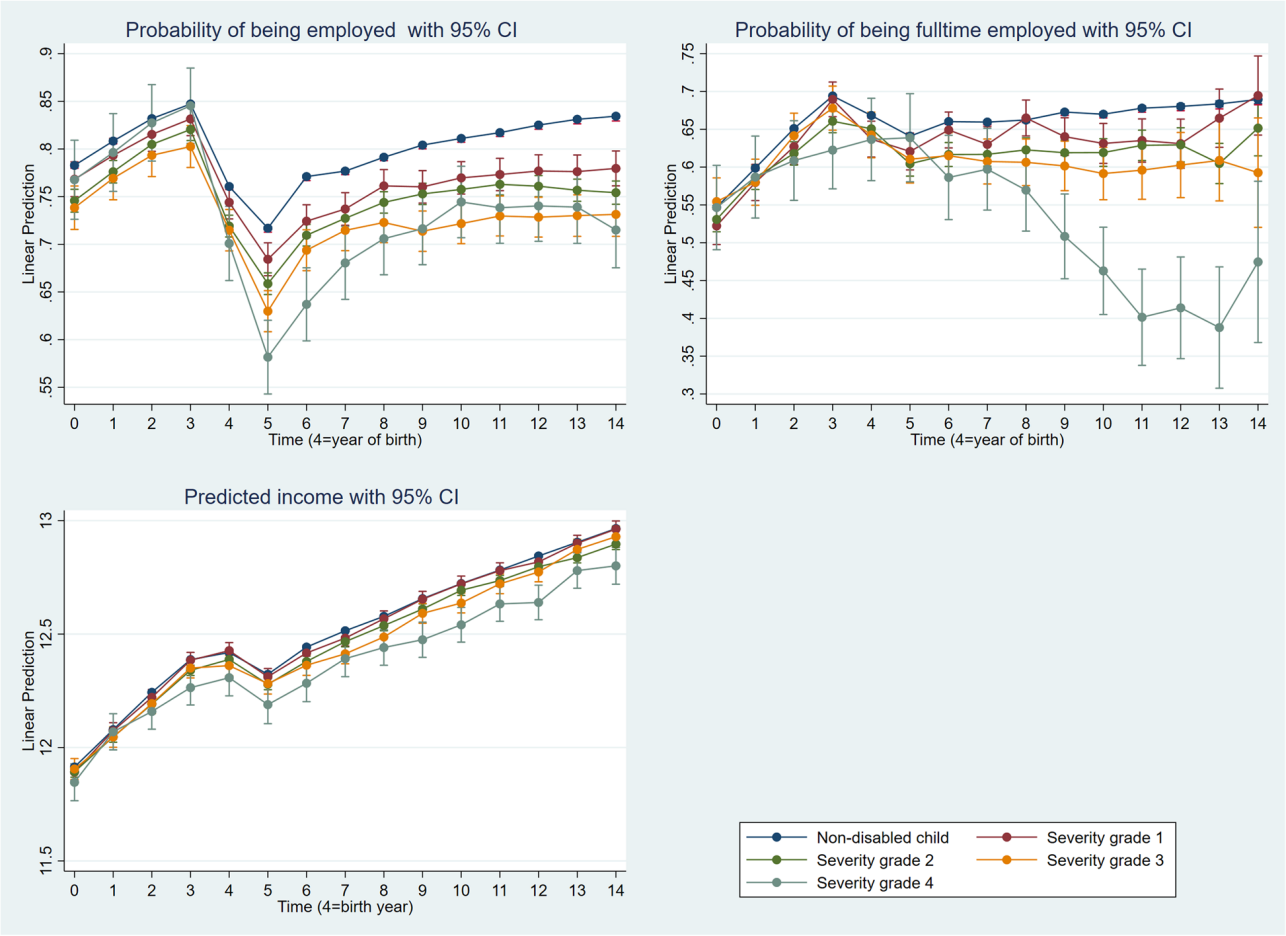
Employment, working hours and labour income (log) among mothers by the severity of the child’s disability. The labour income and working hours analysis are restricted to employed mothers

**Fig. 2:**
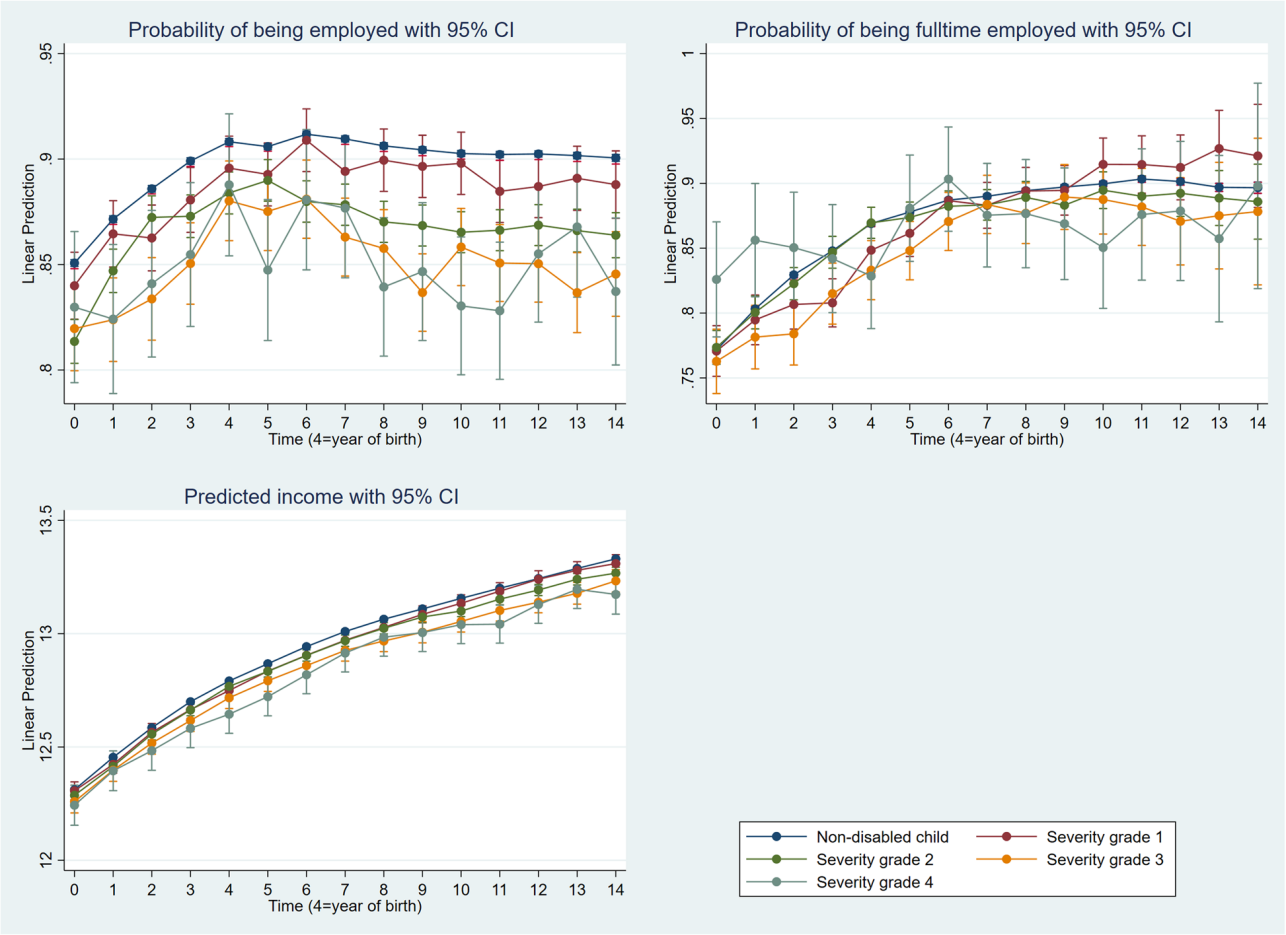
Employment, working hours and labour income (log) among fathers by the severity of the child’s disability. The labour income and working hours analysis are restricted to employed fathers

Statistical analysis was performed using STATA® 17, with the statistical significance level set at *p* < 0.05. The analyses of employment and working hours were performed using a linear probability model (LPM); namely, linear regressions on a binary variable. When the outcome variable is binary, logistic regression is frequently used. However, because the coefficients in logistic regression represent not only the effect of the independent variables but also the extent of unobserved heterogeneity, comparing coefficients across samples is difficult [31]. For that reason, we computed an LPM. This give results in terms of changes in probability. Labour income is the third dependent variable. Ordinary linear regression was used to examine the effect of child disability on parental labour income. The same independent variables were included in the models estimated in the analysis of all outcomes. The results in Figs. 1 and 2 are presented as predicted margins based on the explanatory variables’ means. Examining trends in the employment of parents with children with disabilities can be challenging, as numerous factors aside from having a child with disability can affect employment [2]. Because the present study includes rich longitudinal data with a 14-year dataset before and after the birth of a child for the same individuals, it is possible to examine the effects of raising a child with disability on parental employment.

## Results

Table 1 presents the descriptive statistics of all variables measured at birth. Mothers of children with and without disabilities had average ages at birth of 27 and 28 years, respectively, whereas fathers in the two groups both had average ages at birth of 31. Mothers of children with disabilities constituted 4.2% of all mothers in the observation period, with 0.2% having children at grade 4, 0.7% at grade 3, 2.3% at grade 2, and 1% at grade 1 levels of disability severity. The proportion of fathers of children with disabilities is similar to that of mothers. 86% of mothers of children with disabilities had a majority background, which was higher than those with children without disabilities (82.8%). Fathers of children with disabilities were also more likely to have a majority background. A higher proportion of married persons were in the group of mothers of children without disabilities (31.3%) than mothers of children with disabilities (27.1%). We noticed a similar pattern for fathers. There were differences in educational attainment between the two groups of mothers, which we controlled for in the DiD models. At birth year, mothers of children without disabilities had a higher any college and university attainment than mothers of children with disabilities (38.1% vs. 51.1%). A similar pattern was observed for fathers.

**Table 1:** Descriptive statistics measured at birth for mothers and fathers caring for disabled and non-disabled children

	Mothers		Fathers	
	Child with a disability (*N* = 139,189, 4.2%)	Child without a disability (*N* = 2,816,060, 95.3%)	Child with a disability (*N* = 134,457, 4.2%)	Child without a disability (*N* = 2,725,588, 95.3%)
Age at birth, mean (SD), yrs	27 (5.38)	28 (5.14)	31 (6.29)	31 (6.21)
Employment Status *(%)*				
Not employed	30.1	23.7	13	9.2
Employed	69.9	76.3	87	90.8
Income, log mean (SD)	12.1 (1.1)	12.3 (1.02)	12.6 (0.76)	12.7 (0.72)
Working time *(%)*				
Part time	36.2	31.1	14	12.7
Full time	63.8	68.9	86	87.3
Levels of disability severity *(%)*				
No disability		95.3		95.3
Grade 1	1		1	
Grade 2	2.3		2.3	
Grade 3	0.7		0.7	
Grade 4	0.2		0.2	
Educational level *(%)*				
No education	0.2	0.2	0.2	0.1
Compulsory education	27.3	18.6	26.8	18.9
Upper secondary education	34.4	30.1	44.7	41.9
Any college and university	38.1	51.1	28.3	39.1
Civil status *(%)*				
Married	27.1	31.3	28.8	32.7
Unmarried	72.9	68.7	71.2	67.3
Immigrant background (%)				
Majority	86	82.8	86	84.3
1st generation	13	16.2	13.2	15
2nd generation	1	0.9	0.8	0.7

‘Child with a disability’ = disability severity grades 1–4; measured for primipara mothers

The descriptive statistics results of the time-varying employment measures for 4 years prior and 10 years following birth are presented in Additional file 1: Appendix A, Table S1. Results indicate that mothers of children with disabilities participated less in the labour market. Four years before and 10 years following childbirth, the labour market participation of mothers of children with and without disabilities was (72.7 vs. 79.1) and (74 vs. 82.6), respectively. Ten years after birth, the differences in log mean income between mothers of children with and without disabilities were relatively small (12.3% vs. 12.5%). The results also indicated slightly lower employment participation rates and labour incomes for fathers of children with disabilities than of fathers of children without disabilities. Ten years after birth, mothers of children with disabilities were less likely (63.6%) than mothers of children without disabilities (69.7%) to work full time. In comparison with mothers, the difference in working full time between fathers of children with and without disabilities was substantially smaller (89.7% vs. 90.7%).

The pre-trends in the dependent variables were comparable for treatments (parents of children with disabilities) and controls (parents of children without disabilities), so we assumed that differences between the two groups of parents after the child was born were caused by having a child with disability (Figs. 1 and 2).

### Employment

Controlling for confounders, the analysis of employment (Additional file 1: Appendix B, Table S2: Model 1) shows that mothers of children with disabilities reduced their labour market participation significantly more than mothers of children without disabilities. However, the differences varied with the severity grade of the disability. The effect was more pronounced among mothers who cared for children with more severe disabilities. Among mothers caring for children with a grade 4 disability, the difference amounts to 12 percentage points the year after birth and 10.4 percentage points 10 years after birth. The comparable numbers for mothers caring for children with severity grade 1 were four percentage points ten years after birth. However, the differences one year after birth were quite small and did not attain statistical significance. We noticed that the variation in employment probabilities among mothers did not decrease once a child reached school age; in fact, the effect increased for mothers caring for school-aged children. Figure 1 shows that employment participation gradually increased with time since birth for mothers of children without disabilities. However, for mothers caring for children with disabilities, the pattern was somewhat different: labour market participation increased in the first years after birth for all groups of mothers but flattened out or even decreased (including those whose children had the most severe disability) for mothers of children with disabilities as time since birth passed.

The same regression model for fathers shows that raising children with disabilities had no significant effect on fathers’ employment probabilities (Additional file 1: Appendix B, Table S2: Model 2). There was no statistically significant difference between fathers caring for children with severity grades 1 and 2 and those with children without disabilities (Fig. 2). The pattern in employment probability differences was inconsistent in severity grades 3 and 4. In most years after birth, there was no statistically significant difference between fathers caring for children with severity grades 3 and 4 and those caring for children without disabilities. However, some years did have small but statistically significant differences.

### Working hours

The results in Additional file 1: Appendix C, Table S3: Model 1 show that working hours differed between mothers caring for children with more severe disabilities and those with children without disabilities. In the post-birth period, mothers of children with more severe disabilities tended to shift from full- to part-time employment. The differences became more pronounced as the severity grade increased. In the post-birth period, the difference was not significant for mothers caring for children with severity grade 1 disabilities. The variations at severity grade 2 were small but significant, ranging from two to six percentage points between one year and nine years after birth. In all post-birth years, the differences between comparable groups of mothers were significant at severity grades 3 and 4. Ten years after birth, the difference for mothers caring for children with severity grade 3 was 10.4 percentage points; it was 21.4 percentage points for severity grade 4. Contrary to our expectations, the difference in working hours between comparable groups have increased as children reached school age. Figure 1 shows that, among mothers of children without disabilities, working hours started to decrease in the year after birth, increased again after two years, and then flattened out over time. There is overlap in the confidence intervals between mothers caring for children without disabilities and mothers caring for children with severity grade 1. Working hours for mothers caring for children with disabilities grades 2 and 3 declined in the years after birth but flattened out over time. For disability grade 4, working hours decreased until the child was nine years old.

In general, fathers did not appear to reduce their working hours due to increased care needs (Additional file 1: Appendix C, Table S3: Model 2). A similar pattern is observed in Fig. 2, where the confidence intervals overlap, indicating that the differences in working hours between all groups of fathers were not significant. However, in most years following birth, fathers caring for children with severity grade 4 tended to shift from full- to part-time employment. When the child was 1 year old, the difference in working hours was 5.1 percentage points; it increased to 9.4 percentage points when the child was nine.

### Labour income

The analysis of labour income (Additional file 1: Appendix D, Table S4: Model 1) shows that mothers of children with more severe disabilities earned significantly less than mothers of children without disability. The income disparity also increased as the severity of the child’s disability increases. There was a statistically significant difference in labour income between comparable groups in most years following birth for higher severity levels. Eight years after birth, the difference was 13.7% for mothers caring for children with grade 4 disabilities. The comparable numbers for mothers caring for children with grade 3 and 2 disabilities were 6.1 and 2.6%, respectively. In all years following birth, the differences at severity grade 1 did not reach statistical significance. Counter to our expectations, the differences in labour income between the two groups of mothers increased as their children reached school age. Figure 1 also shows that mothers of children with more severe disabilities earned less than mothers caring for children without disability.

We did not find the same pattern of labour income differences between mothers and fathers. In general, caring for children with disabilities had little impact on fathers’ labour income (Additional file 1: Appendix D, Table S4: Model 2). The income disparity for fathers between comparable groups for grade 1 was not statistically significant in any year after birth. In some years following birth, we found small but significant variations in labour income for fathers caring for children with severity grades 2, 3, and 4; the difference was greater for higher severity grades. Ten years after birth, the labour income of fathers caring for children with a level 4 severity disability was 8.8% lower than among fathers of children without disabilities. The comparable number for fathers caring for children with severity grade 2 was 3.8%. Figure 2 shows that labour income for all groups of fathers gradually increased over time following birth. Fathers of children with more severe disabilities earned the least of all groups of fathers.

## Discussion

This article examined how parental employment, working hours and labour income were affected by caring for a child with disability. We focused on differences in the long-term effects of caring for children with disabilities based on parental gender, disability severity and child age. We expected that, parents of children with disabilities might withdraw from paid employment, reduce their labour income, or shift from stable full-time employment to part-time employment (Table 2). Consistent with previous studies, such as Cantero-Garlito et al. [14], Burton and Phipps [32], Busch and Barry [33], the present study revealed that mothers of children with disabilities were less likely to be employed, worked fewer hours and earned less than mothers of children without disabilities in the post-birth period. Some of the income decline could be the result of mothers shifting from full-time to part-time paid employment after having a child with disability. As expected, the more severe a condition was, the more likely mothers were to reduce working hours or stop working entirely, which is in line with [10, 11, 21, 32, 34, 35]. Mothers of children with less severe disabilities appeared to be affected to a lesser extent.

**Table 2:** Hypothesis testing results

Hypothesis	Result
H1: Parents of children with disabilities are less likely to be employed.	Partially supported
H2: Parents of children with disabilities are less likely to be in full-time employment.	Partially supported
H3: Caring for a child with a disability will result in lower labour earnings.	Supported
H4: The negative employment effects is stronger for mothers than for fathers.	Supported
H5: The negative employment effects is stronger for parents of children with more severe disability.	Supported
H6: The negative employment effects is stronger among parents of pre-school children.	Not supported

Labour market participation, working time, and labour income for fathers of children with less severe disabilities were comparable to that of fathers of children without disabilities. Caring for children with more severe disabilities, on the other hand, reduces fathers’ labour income but had no effect on their working hours or labour market participation. This suggests that after having a child with disability, fathers continued to engage in paid labour and to work full time. However, fathers of children with more severe disabling conditions had less labour income. Such a reduction in income may occur because fathers were not promoted at work but still participated in paid labour and worked full time (but less overtime). This result corresponds with the findings reported by Gunnsteinsson and Steingrimsdottir [13], which indicated that caring for children with disabilities reduce fathers’ income, although the impact is significantly smaller than among mothers.

Contrary to our expectations, the differences in labour market participation and income between mothers of children with and without disabilities increased as their children reached school age. According to Brekke and Nadim [7], part of the reason for this could be the care responsibilities of parents who care for preschool-age children are greater regardless of their disability, which lessens differences in the care burden effect between mothers caring for preschool-age children with disabilities and those caring for children without disabilities of the same age.

Our study provides evidence that mothers caring for children with more severe disabilities significantly reduce their participation in and income from employment. It may be that these mothers could have participated more in working life if they received more robust welfare support. The expected drop in income could be attributed to the insufficiency of government benefits to fully offset the loss in earnings among mothers. Additionally, our results show that mothers caring for children with less severe disabling conditions appear to remain in the workforce. This trend suggests that the Norwegian welfare state’s generous family policy may indeed be helping to address challenges related to work–family balance among this group of mothers. As Brekke and Nadim [7] also suggest, Norway’s universal healthcare coverage may be serving to address health problems associated with mothers’ caregiving responsibilities and prevent them from losing paid employment entirely. Easy access to sick leave could also help mothers remain in the workforce longer than they would in other national contexts.

Most prior studies on the effects of caring for children with disabilities have been cross-sectional which cannot account for unobserved heterogeneity that may influence both child disability and parents’ labor market participation. The use of the quasi-experimental DiD design in the present study helps to overcome this limitation. Some of the longitudinal studies, including Brekke and Nadim [7], Reisel et al. [36], examined only the employment effects in the first few years following birth. Using rich register data, our study adds to the literature by investigating the long-term effects of caring for children with disabilities prior to birth and for the first 10 years of their lives. Our study included all children in Norway who receive attendance benefits, which responds to concerns about selection bias affecting a study’s validity. Another strength is that the study included register data on labour market participation, working hours and labour income, which enabled us to examine whether the employment effect is comparable between those variables. From our results on working hours and labour income, we can conclude that the observed reduction in labour income among mothers of children with disabilities was due to their spending fewer hours in paid labour.

Because direct measures in the registry data were unavailable, we used a proxy measure for severity, which limited the study’s capacity to determine which types of disability have greater employment effects than others. There is also a possible challenge with using attendance benefits due to concerns about the extent to which children with disabilities are captured in those data. However, according to Wendelborg and Tøssebro [37], 91% of surveyed Norwegian parents of children with disabilities received attendance benefits, indicating that using attendance benefits is appropriate to identify families caring for children with disabilities. By including information on the rate and extent of attendance benefits, we can obtain an understanding of a child’s level of need for assistance. Despite these limitations, the severity proxy showed that mothers of children with more severe disabilities were the most affected mothers, in accordance with the previous research cited above.

## Conclusion

Caring for children with disabilities has a negative effect on mothers’ labour market participation, working hours and labour income. The employment effect is more pronounced among mothers of children with more severe disability. Although their labour income is lower, employment probabilities and working hours of fathers of children with more severe disabilities remain consistent in the post-birth period. This may indicate that, even in an equality-promoting welfare state like Norway, mothers continue to bear the primary responsibilities for caregiving. Our findings suggest that policymakers and child welfare stakeholders should evaluate policy options and provide the necessary welfare support, particularly for mothers caring for children with a more severe disability, in order to improve their work–family balance, with an emphasis on addressing health risks that may prevent them from participating in the workforce. It is important to assess mothers’ working environments to see whether they have an adverse effect on their health, resulting in job loss. Therefore, future research may investigate the occupational health risks associated with caring for children with disabilities.

## Supplementary Information

**Additional file 1: Table S1.** Employment characteristics for mothers caring for children with and without disabilities, 4 years before and 10 years after birth. **Table S2.** Employment. Dependent variable: employment status in reference week. Linear probability model, mothers and fathers. The sample is from birth cohorts 2004–2011, primipara. **Table S3.** Working time. Dependent variable: part-time vs full-time work. Linear probability model employed mothers and fathers. The sample is from birth cohorts 2004–2011, primipara. **Table S4.** Labour Income, Dependent variable: income (log) from employment, OLS regression analyses, employed mothers and fathers. The sample is birth cohort 2004-2011, primipara.

## Data Availability

The data used in this study are available from the Statistic Norway, but they are not publicly accessible because they were used under license for this study. The data may, however, be accessible from the authors upon reasonable request and with permission of the Statistic Norway.

## Abbreviations

DiD: Difference-in-differences
FD-Trygd: Historical event database
NAV: Norwegian Labour and Welfare Administration
SSB: Statistics Norway
P: Probability value
SD: Standard deviations
