# Supplementary material for: Impact of child disability on parental employment and labour income: a quasi-experimental study of parents of children with disabilities in Norway
Source: BMC Public Health. 2022 Sep 24;22:1813. doi: 10.1186/s12889-022-14195-5 (PMC9508753; doi:10.1186/s12889-022-14195-5)
Supplement: Supplementary file 1 — Additional file 1: Table S1. Employment characteristics for mothers caring for children with and without disabilities, 4 years before and 10 years after birth. Table S2. Employment. Dependent variable: employment status in reference week. Linear probability model, mothers and fathers. The sample is from birth cohorts 2004–2011, primipara. Table S3. Working time. Dependent variable: part-time vs full-time work. Linear probability model employed mothers and fathers. The sample is from birth cohorts 2004–2011, primipara. Table S4. Labour Income, Dependent variable: income (log) from employment, OLS regression analyses, employed mothers and fathers. The sample is birth cohort 2004-2011, primipara. [file 12889_2022_14195_MOESM1_ESM.docx]

**Appendix A**

**Table S1** Employment characteristics for mothers caring for children with and without disabilities, 4 years before and 10 years after birth

|  | 4 years before birth | | 10 years after birth | |
| --- | --- | --- | --- | --- |
|  | Child with a disability | Child without a disability | Child with a disability | Child without a disability |
| **Mothers** |  |  |  |  |
| Employment Status *(%)* |  |  |  |  |
| Not employed | 27.3 | 20.9 | 26 | 17.4 |
| Employed | 72.7 | 79.1 | 74 | 82.6 |
| Income, log mean (SD) | 11.5  (1.2) | 11.7  (1.1) | 12.3  (2.2) | 12.5  (2.3) |
| Working time *(%)* |  |  |  |  |
| Part time | 47.6 | 44.1 | 36.4 | 30.3 |
| Full time | 52.4 | 55.9 | 63.6 | 69.7 |
| **Fathers** |  |  |  |  |
| Employment Status *(%)* |  |  |  |  |
| Not employed | 19.7 | 15.1 | 14.2 | 9.9 |
| Employed | 80.3 | 84.9 | 88.8 | 90.1 |
| Income, log mean (SD) | 12  (1.1) | 12.2  (1.1) | 12.9  (1.9) | 13.1  (1.6) |
| Working time *(%)* |  |  |  |  |
| Part time | 21.6 | 21.6 | 10.2 | 9.3 |
| Full time | 78.4 | 78.4 | 89.7 | 90.7 |

*Notes*: ‘Child with a disability’ = disability severity grades 1-4; measured for primipara mothers

**Appendix B**

**Table S2** Employment. Dependent variable: employment status in reference week. Linear probability model, mothers and fathers. The sample is from birth cohorts 2004–2011, primipara.

|  | Mothers (model 1) | | Fathers (model 2) | |
| --- | --- | --- | --- | --- |
| Variables | Coeff | St.error | Coeff | St.error |
| Caring for a disabled child |  |  |  |  |
| Time (ref: 3-year prior birth) |  |  |  |  |
| 3 years prior birth | 0.025** | 0.001 | 0.020** | 0.001 |
| 2 years prior birth | 0.049** | 0.001 | 0.035** | 0.001 |
| 1 year prior birth | 0.064** | 0.001 | 0.048** | 0.001 |
| Birth year | -0.022** | 0.001 | 0.057** | 0.001 |
| 1 year after birth | -0.065** | 0.001 | 0.055** | 0.001 |
| 2 years after birth | -0.011** | 0.001 | 0.061** | 0.001 |
| 3 years after birth | -0.005** | 0.001 | 0.058** | 0.001 |
| 4 years after birth | 0.008** | 0.001 | 0.055** | 0.001 |
| 5 years after birth | 0.021** | 0.001 | 0.053** | 0.001 |
| 6 years after birth | 0.282** | 0.001 | 0.051** | 0.001 |
| 7 years after birth | 0.343** | 0.001 | 0.051** | 0.001 |
| 8 years after birth | 0.042** | 0.001 | 0.051** | 0.001 |
| 9 years after birth | 0.048** | 0.001 | 0.050** | 0.001 |
| 10 years after birth | 0.051** | 0.001 | 0.049** | 0.001 |
| Interactions (Severity grade 1) |  |  |  |  |
| 3 years prior birth x disabled child | -0.005 | 0.10 | 0.003 | 0.010 |
| 2 years prior birth x disabled child | -0.002 | 0.10 | -0.012 | 0.009 |
| 1 year prior birth x disabled child | -0.001 | 0.10 | -.0007 | 0.009 |
| Birth year x disabled child | -0.002 | 0.10 | -.0001 | 0.009 |
| 1 year after birth x disabled child | -0.018 | 0.10 | -0.002 | 0.009 |
| 2 years after birth x disabled child | -0.032** | 0.10 | 0.007 | 0.009 |
| 3 years after birth x disabled child | -0.025* | 0.10 | -0.004 | 0.009 |
| 4 years after birth x disabled child | -0.015 | 0.10 | 0.003 | 0.009 |
| 5 years after birth x disabled child | -0.029** | 0.10 | -0.002 | 0.009 |
| 6 years after birth x disabled child | -0.026* | 0.01 | -0.006 | 0.009 |
| 7 years after birth x disabled child | -0.029** | 0.01 | -0.006 | 0.009 |
| 8 years after birth x disabled child | -0.033** | 0.10 | -0.004 | 0.009 |
| 9 years after birth x disabled child | -0.040** | 0.10 | -0.001 | 0.009 |
| 10 years after birth x disabled child | -0.040** | 0.11 | -0.001 | 0.009 |
| Interactions (Severity grade 2) |  |  |  |  |
| 3 years prior birth x disabled child | 0.004 | 0.017 | 0.003 | 0.010 |
| 2 years prior birth x disabled child | 0.010 | 0.007 | -0.012 | 0.009 |
| 1 year prior birth x disabled child | 0.010 | 0.007 | -.0007 | 0.009 |
| Birth year x disabled child | -0.004 | 0.007 | -.0001 | 0.009 |
| 1 year after birth x disabled child | -0.021** | 0.007 | -0.002 | 0.009 |
| 2 years after birth x disabled child | -0.024** | 0.007 | 0.007 | 0.009 |
| 3 years after birth x disabled child | -0.012 | 0.007 | -0.004 | 0.009 |
| 4 years after birth x disabled child | -0.010 | 0.006 | 0.003 | 0.009 |
| 5 years after birth x disabled child | -0.014* | 0.006 | -0.002 | 0.009 |
| 6 years after birth x disabled child | -0.016* | 0.006 | -0.006 | 0.009 |
| 7 years after birth x disabled child | -0.017* | 0.006 | -0.006 | 0.009 |
| 8 years after birth x disabled child | -0.027** | 0.006 | -0.004 | 0.009 |
| 9 years after birth | -0.037** | 0.007 | 0.050** | 0.001 |
| 10 years after birth | -0.043** | 0.007 | 0.049** | 0.001 |
| Interactions (Severity grade 3) |  |  |  |  |
| 3 years prior birth x disabled child | 0.005 | 0.013 | -0.016 | 0.011 |
| 2 years prior birth x disabled child | 0.006 | 0.013 | -0.021 | 0.011 |
| 1 year prior birth x disabled child | -0.001 | 0.013 | -.0017 | 0.011 |
| Birth year x disabled child | -0.001 | 0.013 | -.0001 | 0.011 |
| 1 year after birth x disabled child | -0.042** | 0.013 | -0.001 | 0.011 |
| 2 years after birth x disabled child | -0.032* | 0.013 | -0.001 | 0.011 |
| 3 years after birth x disabled child | -0.017 | 0.013 | -0.015 | 0.011 |
| 4 years after birth x disabled child | -0.024 | 0.013 | -0.017 | 0.011 |
| 5 years after birth x disabled child | -0.045** | 0.013 | -0.036** | 0.011 |
| 6 years after birth x disabled child | -0.044** | 0.013 | -0.013 | 0.011 |
| 7 years after birth x disabled child | -0.043** | 0.013 | -0.020 | 0.011 |
| 8 years after birth x disabled child | -0.052** | 0.013 | -0.020 | 0.011 |
| 9 years after birth | -0.056** | 0.013 | -0.033** | 0.011 |
| 10 years after birth | -0.058** | 0.014 | -0.023 | 0.012 |
| Interactions (Severity grade 4) |  |  |  |  |
| 3 years prior birth x disabled child | 0.003 | 0.024 | -0.026 | 0.021 |
| 2 years prior birth x disabled child | 0.010 | 0.024 | -0.024 | 0.021 |
| 1 year prior birth x disabled child | 0.013 | 0.024 | -.0023 | 0.020 |
| Birth year x disabled child | -0.044 | 0.024 | -.0001 | 0.020 |
| 1 year after birth x disabled child | -0.12** | 0.024 | -0.037 | 0.020 |
| 2 years after birth x disabled child | -0.11** | 0.024 | -0.010 | 0.020 |
| 3 years after birth x disabled child | -0.081** | 0.024 | -0.011 | 0.020 |
| 4 years after birth x disabled child | -0.07** | 0.024 | -0.046* | 0.020 |
| 5 years after birth x disabled child | -0.072** | 0.023 | -0.036 | 0.020 |
| 6 years after birth x disabled child | -0.051** | 0.023 | -0.051** | 0.020 |
| 7 years after birth x disabled child | -0.063** | 0.023 | -0.053* | 0.020 |
| 8 years after birth x disabled child | -0.069** | 0.023 | -0.026 | 0.020 |
| 9 years after birth | -0.077** | 0.024 | -0.012 | 0.020 |
| 10 years after birth | -0.10** | 0.024 | -0.042* | 0.020 |
| Constant | 0.299* | 0.008 | 0.706** | 0.021 |
| N (person-years) | 2,827,576 |  | *2,383,326* |  |

*Note:* age at birth, immigrant background, educational level, marital status, number of children in the household and birth cohort are included in the model.

**Appendix C**

**Table S3** Working time. Dependent variable: part-time vs full-time work. Linear probability model employed mothers and fathers. The sample is from birth cohorts 2004–2011, primipara.

|  | Mothers (model 1) | | Fathers (model 2) | |
| --- | --- | --- | --- | --- |
| Variables | Coeff | St.error | Coeff | St.error |
| Caring for a disabled child |  |  |  |  |
| Time (ref: 3-year prior birth) |  |  |  |  |
| 3 years prior birth | 0.051** | 0.001 | 0.031** | 0.001 |
| 2 years prior birth | 0.104** | 0.001 | 0.057** | 0.001 |
| 1 year prior birth | 0.147** | 0.001 | 0.076** | 0.001 |
| Birth year | 0.121** | 0.001 | 0.097** | 0.001 |
| 1 year after birth | 0.093** | 0.001 | 0.106** | 0.001 |
| 2 years after birth | 0.113** | 0.001 | 0.115** | 0.001 |
| 3 years after birth | 0.112** | 0.001 | 0.118** | 0.001 |
| 4 years after birth | 0.115** | 0.001 | 0.122** | 0.001 |
| 5 years after birth | 0.125** | 0.001 | 0.125** | 0.001 |
| 6 years after birth | 0.123** | 0.001 | 0.128** | 0.001 |
| 7 years after birth | 0.131** | 0.001 | 0.131** | 0.001 |
| 8 years after birth | 0.133** | 0.002 | 0.129** | 0.001 |
| 9 years after birth | 0.136** | 0.002 | 0.125** | 0.001 |
| 10 years after birth | 0.142** | 0.003 | 0.125** | 0.002 |
| Interactions (Severity grade 1) |  |  |  |  |
| 3 years prior birth x disabled child | 0.005 | 0.015 | -0.008 | 0.012 |
| 2 years prior birth x disabled child | 0.001 | 0.015 | -0.022 | 0.012 |
| 1 year prior birth x disabled child | 0.02 | 0.015 | -0.039 | 0.012 |
| Birth year x disabled child | -0.007 | 0.015 | -0.02 | 0.012 |
| 1 year after birth x disabled child | 0.004 | 0.015 | -0.016 | 0.011 |
| 2 years after birth x disabled child | 0.014 | 0.015 | 0.001 | 0.011 |
| 3 years after birth x disabled child | -0.005 | 0.015 | -0.006 | 0.011 |
| 4 years after birth x disabled child | 0.027 | 0.015 | 0.001 | 0.012 |
| 5 years after birth x disabled child | -0.008 | 0.015 | -0.002 | 0.012 |
| 6 years after birth x disabled child | -0.014 | 0.016 | 0.016 | 0.012 |
| 7 years after birth x disabled child | -0.018 | 0.017 | 0.012 | 0.013 |
| 8 years after birth x disabled child | -0.025 | 0.019 | 0.012 | 0.015 |
| 9 years after birth x disabled child | 0.006 | 0.022 | 0.031 | 0.017 |
| 10 years after birth x disabled child | 0.03 | 0.028 | 0.025 | 0.022 |
| Interactions (Severity grade 2) |  |  |  |  |
| 3 years prior birth x disabled child | 0.003 | 0.009 | -0.004 | 0.007 |
| 2 years prior birth x disabled child | -0.001 | 0.009 | -0.008 | 0.007 |
| 1 year prior birth x disabled child | -0.017 | 0.009 | -0.003 | 0.007 |
| Birth year x disabled child | -0.014 | 0.009 | -0.015 | 0.007 |
| 1 year after birth x disabled child | -0.020* | 0.009 | -0.006 | 0.007 |
| 2 years after birth x disabled child | -0.027** | 0.009 | -0.007 | 0.007 |
| 3 years after birth x disabled child | -0.026** | 0.009 | -0.016 | 0.008 |
| 4 years after birth x disabled child | -0.023* | 0.010 | 0.007 | 0.008 |
| 5 years after birth x disabled child | -0.037** | 0.010 | -0.015 | 0.008 |
| 6 years after birth x disabled child | -0.034** | 0.011 | -0.069 | 0.008 |
| 7 years after birth x disabled child | -0.033** | 0.011 | -0.015 | 0.009 |
| 8 years after birth x disabled child | -0.034** | 0.013 | -0.011 | 0.010 |
| 9 years after birth | -0.062** | 0.014 | -0.010 | 0.011 |
| 10 years after birth | -0.021 | 0.019 | -0.012 | 0.015 |
| Interactions (Severity grade 3) |  |  |  |  |
| 3 years prior birth x disabled child | -0.026 | 0.018 | -0.012 | 0.015 |
| 2 years prior birth x disabled child | -0.016 | 0.018 | -0.036 | 0.015 |
| 1 year prior birth x disabled child | -0.023 | 0.018 | -.0024 | 0.014 |
| Birth year x disabled child | -0.033* | 0.018 | -.0027 | 0.014 |
| 1 year after birth x disabled child | -0.037 | 0.019 | -0.021 | 0.014 |
| 2 years after birth x disabled child | -0.052** | 0.019 | -0.007 | 0.014 |
| 3 years after birth x disabled child | -0.059** | 0.018 | -0.002 | 0.014 |
| 4 years after birth x disabled child | -0.063** | 0.019 | -0.008 | 0.014 |
| 5 years after birth x disabled child | -0.078* | 0.020 | 0.001 | 0.015 |
| 6 years after birth x disabled child | -0.085** | 0.021 | -0.033 | 0.016 |
| 7 years after birth x disabled child | -0.089* | 0.022 | -0.012 | 0.017 |
| 8 years after birth x disabled child | -0.085** | 0.024 | -0.021 | 0.019 |
| 9 years after birth | -0.082** | 0.029 | -0.013 | 0.022 |
| 10 years after birth | -0.10** | 0.038 | -0.009 | 0.030 |
| Interactions (Severity grade 4) |  |  |  |  |
| 3 years prior birth x disabled child | -0.011 | 0.033 | -0.014 | 0.027 |
| 2 years prior birth x disabled child | -0.041 | 0.033 | -0.033 | 0.026 |
| 1 year prior birth x disabled child | -0.071* | 0.032 | -.0060* | 0.026 |
| Birth year x disabled child | -0.031 | 0.034 | -.0094** | 0.025 |
| 1 year after birth x disabled child | -0.016 | 0.035 | -0.051* | 0.026 |
| 2 years after birth x disabled child | -0.073* | 0.034 | -0.038 | 0.025 |
| 3 years after birth x disabled child | -0.061 | 0.034 | -0.069** | 0.025 |
| 4 years after birth x disabled child | -0.092** | 0.034 | -0.072** | 0.026 |
| 5 years after birth x disabled child | -0.163** | 0.034 | -0.082** | 0.027 |
| 6 years after birth x disabled child | -0.206** | 0.035 | -0.10** | 0.028 |
| 7 years after birth x disabled child | -0.276** | 0.038 | -0.081 | 0.030 |
| 8 years after birth x disabled child | -0.265** | 0.039 | -0.077* | 0.031 |
| 9 years after birth | -0.295** | 0.045 | -0.094* | 0.036 |
| 10 years after birth | -0.214** | 0.058 | -0.053 | 0.043 |
| Constant | 0.114** | 0.008 | 0.562** | 0.009 |
| N (person-years) | 1,736,071 |  | 1,644,560 |  |

*Note:* age at birth, immigrant background, educational level, marital status, number of children in the household and birth cohort are included in the model.

**Appendix D**

**Table S4** Labour Income, Dependent variable: income (log) from employment, OLS regression analyses, employed mothers and fathers. The sample is birth cohort 2004-2011, primipara.

|  | Mothers (model 1) | | Fathers (model 2) | |
| --- | --- | --- | --- | --- |
| Variables | Coeff | St.error | Coeff | St.error |
| Caring for a disabled child |  |  |  |  |
| Time (ref: 3-year prior birth) |  |  |  |  |
| 3 years prior birth | 0.164** | 0.001 | 0.143** | 0.001 |
| 2 years prior birth | 0.328** | 0.001 | 0.273** | 0.001 |
| 1 year prior birth | 0.473** | 0.001 | 0.388** | 0.001 |
| Birth year | 0.504** | 0.001 | 0.479** | 0.001 |
| 1 year after birth | 0.408** | 0.001 | 0.555** | 0.001 |
| 2 years after birth | 0.528** | 0.001 | 0.631** | 0.001 |
| 3 years after birth | 0.600** | 0.001 | 0.698** | 0.001 |
| 4 years after birth | 0.633** | 0.001 | 0.752** | 0.001 |
| 5 years after birth | 0.741** | 0.001 | 0.797** | 0.001 |
| 6 years after birth | 0.808** | 0.001 | 0.844** | 0.001 |
| 7 years after birth | 0.867** | 0.001 | 0.899** | 0.001 |
| 8 years after birth | 0.929** | 0.002 | 0.931** | 0.001 |
| 9 years after birth | 0.990** | 0.002 | 0.976** | 0.001 |
| 10 years after birth | 1.050** | 0.002 | 1.018** | 0.001 |
| Interactions (Severity grade 1) |  |  |  |  |
| 3 years prior birth x disabled child | 0.017 | 0.018 | -0.026 | 0.018 |
| 2 years prior birth x disabled child | 0.002 | 0.018 | -0.015 | 0.018 |
| 1 year prior birth x disabled child | 0.020 | 0.018 | -0.030 | 0.017 |
| Birth year x disabled child | 0.030 | 0.018 | -0.374 | 0.017 |
| 1 year after birth x disabled child | 0.011 | 0.018 | -0.027 | 0.017 |
| 2 years after birth x disabled child | -0.003 | 0.018 | -0.033 | 0.017 |
| 3 years after birth x disabled child | -0.009 | 0.018 | -0.033 | 0.017 |
| 4 years after birth x disabled child | 0.011 | 0.018 | -0.031 | 0.017 |
| 5 years after birth x disabled child | 0.204 | 0.018 | -0.019 | 0.017 |
| 6 years after birth x disabled child | 0.021 | 0.018 | -0.016 | 0.017 |
| 7 years after birth x disabled child | 0.020 | 0.018 | -0.008 | 0.017 |
| 8 years after birth x disabled child | -0.003 | 0.018 | 0.002 | 0.017 |
| 9 years after birth x disabled child | 0.018 | 0.018 | -0.003 | 0.017 |
| 10 years after birth x disabled child | 0.020 | 0.019 | -0.015 | 0.018 |
| Interactions (Severity grade 2) |  |  |  |  |
| 3 years prior birth x disabled child | -0.010 | 0.012 | -0.015 | 0.012 |
| 2 years prior birth x disabled child | -0.030 | 0.012 | -0.003 | 0.012 |
| 1 year prior birth x disabled child | -0.025 | 0.012 | -0.012 | 0.011 |
| Birth year x disabled child | -0.009 | 0.012 | 0.006 | 0.011 |
| 1 year after birth x disabled child | -0.023* | 0.012 | -0.008 | 0.011 |
| 2 years after birth x disabled child | -0.042** | 0.012 | -0.014 | 0.011 |
| 3 years after birth x disabled child | -0.026* | 0.012 | -0.016 | 0.011 |
| 4 years after birth x disabled child | -0.019 | 0.012 | -0.015 | 0.011 |
| 5 years after birth x disabled child | -0.024* | 0.012 | -0.010 | 0.011 |
| 6 years after birth x disabled child | -0.008* | 0.012 | -0.031** | 0.011 |
| 7 years after birth x disabled child | -0.025* | 0.012 | -0.023 | 0.011 |
| 8 years after birth x disabled child | -0.026* | 0.012 | -0.025* | 0.011 |
| 9 years after birth | -0.046** | 0.012 | -0.023 | 0.012 |
| 10 years after birth | -0.047** | 0.013 | -0.038** | 0.012 |
| Interactions (Severity grade 3) |  |  |  |  |
| 3 years prior birth x disabled child | -0.023 | 0.023 | -0.004 | 0.023 |
| 2 years prior birth x disabled child | -0.040 | 0.023 | -0.015 | 0.023 |
| 1 year prior birth x disabled child | -0.028 | 0.023 | -.0030 | 0.022 |
| Birth year x disabled child | -0.049* | 0.023 | -.0021 | 0.022 |
| 1 year after birth x disabled child | -0.032 | 0.023 | -0.022 | 0.022 |
| 2 years after birth x disabled child | -0.071** | 0.023 | -0.031 | 0.022 |
| 3 years after birth x disabled child | -0.092** | 0.023 | -0.031 | 0.022 |
| 4 years after birth x disabled child | -0.082** | 0.023 | -0.043 | 0.022 |
| 5 years after birth x disabled child | -0.055* | 0.023 | 0.050* | 0.022 |
| 6 years after birth x disabled child | -0.076** | 0.023 | -0.049* | 0.022 |
| 7 years after birth x disabled child | -0.051* | 0.023 | -0.045* | 0.022 |
| 8 years after birth x disabled child | -0.061** | 0.023 | -0.051* | 0.022 |
| 9 years after birth | -0.022 | 0.024 | -0.057* | 0.022 |
| 10 years after birth | -0.026 | 0.025 | -0.044 | 0.023 |
| Interactions (Severity grade 4) |  |  |  |  |
| 3 years prior birth x disabled child | 0.057 | 0.042 | 0.008 | 0.041 |
| 2 years prior birth x disabled child | -0.016 | 0.041 | -0.032 | 0.040 |
| 1 year prior birth x disabled child | -0.055 | 0.041 | -.0049 | 0.040 |
| Birth year x disabled child | -0.043 | 0.043 | -.0078* | 0.039 |
| 1 year after birth x disabled child | -0.066 | 0.044 | -0.076 | 0.039 |
| 2 years after birth x disabled child | -0.091* | 0.043 | -0.055 | 0.039 |
| 3 years after birth x disabled child | -0.055 | 0.042 | -0.026 | 0.039 |
| 4 years after birth x disabled child | -0.069** | 0.042 | -0.010 | 0.039 |
| 5 years after birth x disabled child | -0.113** | 0.042 | -0.036 | 0.039 |
| 6 years after birth x disabled child | -0.114** | 0.042 | -0.047 | 0.039 |
| 7 years after birth x disabled child | -0.081* | 0.041 | -0.090* | 0.039 |
| 8 years after birth x disabled child | -0.137** | 0.041 | -0.046 | 0.039 |
| 9 years after birth | -0.057 | 0.042 | -0.023 | 0.039 |
| 10 years after birth | -0.096* | 0.043 | -0.088* | 0.040 |
| Constant | 10.18** | 0.008 | 11.44** | 0.041 |
| N (person-years) | 2,275,028 |  | 2,143,169 |  |

*Note:* age at birth, immigrant background, educational level, marital status, number of children in the household and birth cohort are included in the model.
